# Mechanisms for human genomic rearrangements

**DOI:** 10.1186/1755-8417-1-4

**Published:** 2008-11-03

**Authors:** Wenli Gu, Feng Zhang, James R Lupski

**Affiliations:** 1 Department of Molecular and Human Genetics, Baylor College of Medicine, Houston, TX 77030, USA; 2 Department of Pediatrics, Baylor College of Medicine, Houston, TX 77030, USA; 3 Texas Children's Hospital, Houston, TX 77030, USA; 4 Institute of Human Genetics, Ludwig-Maximilians-University, School of Medicine, Munich 80336, Germany

## Abstract

Genomic rearrangements describe gross DNA changes of the size ranging from a couple of hundred base pairs, the size of an average exon, to megabases (Mb). When greater than 3 to 5 Mb, such changes are usually visible microscopically by chromosome studies. Human diseases that result from genomic rearrangements have been called genomic disorders. Three major mechanisms have been proposed for genomic rearrangements in the human genome. Non-allelic homologous recombination (NAHR) is mostly mediated by low-copy repeats (LCRs) with recombination hotspots, gene conversion and apparent minimal efficient processing segments. NAHR accounts for most of the recurrent rearrangements: those that share a common size, show clustering of breakpoints, and recur in multiple individuals. Non-recurrent rearrangements are of different sizes in each patient, but may share a smallest region of overlap whose change in copy number may result in shared clinical features among different patients. LCRs do not mediate, but may stimulate non-recurrent events. Some rare NAHRs can also be mediated by highly homologous repetitive sequences (for example, *Alu*, LINE); these NAHRs account for some of the non-recurrent rearrangements. Other non-recurrent rearrangements can be explained by non-homologous end-joining (NHEJ) and the Fork Stalling and Template Switching (FoSTeS) models. These mechanisms occur both in germ cells, where the rearrangements can be associated with genomic disorders, and in somatic cells in which such genomic rearrangements can cause disorders such as cancer. NAHR, NHEJ and FoSTeS probably account for the majority of genomic rearrangements in our genome and the frequency distribution of the three at a given locus may partially reflect the genomic architecture in proximity to that locus. We provide a review of the current understanding of these three models.

## Introduction

Genomic rearrangements describe mutational changes in the genome such as duplication, deletion, insertion, inversion, and translocation that are different from the traditional Watson-Crick base pair alterations [1]. Genomic rearrangements can represent polymorphisms that are neutral in function, or they can also convey phenotypes via diverse mechanisms, including changing the copy number (that is, copy number variation or CNV) of dosage-sensitive genes, disrupting genes, creating fusion genes or other mechanisms (reviewed in [1]). The pathological conditions caused by genomic rearrangements are collectively defined as genomic disorders [1-3].

**Figure 1:**
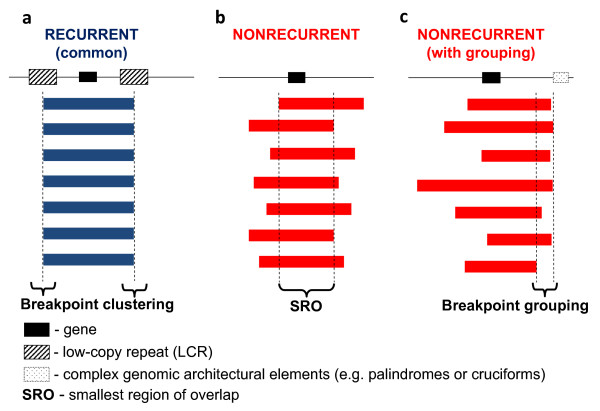
**Experimental observations of recurrent and non-recurrent genomic rearrangements associated with genomic disorders**. The long thin line signifies the genomic region undergoing genomic rearrangements. The black rectangle depicts a gene which is located in the rearranged region and can be affected by the rearrangements. The thick blue (in **a**) and red (in **b **and **c**) bars represent the rearrangements (duplications, deletions or inversions) with their breakpoints **a**. recurrent rearrangements with a common size and clustered breakpoints. Most of the recurrent rearrangements result from non-allelic homologous recombination (NAHR). The two hatched rectangles flanking the gene depict the low-copy repeats (LCRs) functioning as substrates for NAHR. The rearrangement breakpoints are clustered inside the LCRs. **b**. Non-recurrent rearrangements. The breakpoints of the non-recurrent rearrangements are scattered in the genomic region. Note, that all of the rearrangements share a common genomic region of overlap, the smallest region of overlap, that encompasses a gene necessary for the conveyed phenotypic trait, which enables these rearrangements to be ascertained. **c**. Non-recurrent rearrangements with grouping of one breakpoint. Some of the non-recurrent rearrangements have one of their breakpoints localized in one small genomic region. This grouping of breakpoints is distinct from breakpoint clustering, but like clustering, it may reflect underlying genomic architecture (for example, palindrome or cruciform) important to the rearrangement mechanism, depicted as the dotted rectangle in Figure **1c**.

Typically, the term 'genomic rearrangements' is only used to describe gross DNA changes ranging from thousands to sometimes millions of base pairs that can cover clusters of different genes [1]. Genomic rearrangements of this size have been considered to be clearly distinct from the small-scale gene mutations (for example, point mutations, indels) regarding not only the size of the rearranged DNA but also the underlying mechanisms for both the formation of the rearrangements and the conveying of phenotypes (that is, mechanisms upstream and downstream of the rearrangements). Monogenic point mutations usually reflect errors of DNA replication and/or repair [1,2], whereas the gross genomic rearrangements are often caused by other mechanisms mediated or stimulated by genomic structural features (that is, genomic architecture) [1]. Disease-causing genomic rearrangements can be recurrent, with a common size and fixed breakpoints (that is, breakpoints cluster); or non-recurrent with different sizes and distinct breakpoints for each event. The non-recurrent rearrangements share a common genomic region-of-overlap, the smallest region of overlap (SRO), that encompasses the locus associated with the conveyed genomic disorder (Figure 1).

Three major mechanisms have been proposed for genomic rearrangements in the human genome: non-allelic homologous recombination (NAHR), non-homologous end-joining (NHEJ) and the Fork Stalling and Template Switching (FoSTeS) models.

## Recurrent genomic rearrangements are caused by NAHR

### 1. NAHR occurs preferentially at the so-called 'hotspots' inside low-copy repeats

A large number of DNA rearrangements of the same genomic interval have been observed in different individuals, that is they have a recurrent nature [1]. Most recurrent genomic rearrangements are caused by NAHR between two low-copy repeats (LCRs, also called segmental duplications, SD) [4,5]. LCRs are region-specific DNA blocks usually of 10 to 300 kilobase (kb) in size and of > 95% to 97% similarity to each other [5,6]. Bailey and Eichler recently reviewed the distribution and evolution of mammalian LCRs (referred to as SD therein) [6].

**Figure 2:**
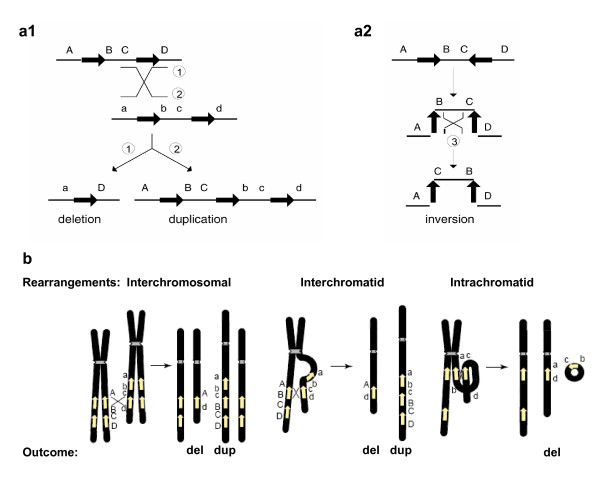
**Genomic rearrangements (Adapted from **[2]** and **[5]**)**. **a1 **and **a2 **Genomic rearrangements resulting from recombination between low-copy repeats (LCRs). LCRs are depicted as black arrows with the orientation indicated by the direction of the arrowhead. Capital letters above the thin horizontal lines refer to the flanking unique sequences (for example, A). Homologues on the other strand (can be another chromatid or the homologous chromosome) are also shown (for example, a). Thin diagonal lines refer to a recombination event with the results shown by numbers 1, 2 and 3. **a1 **Recombination between direct repeats results in deletion and/or duplication. **a2 **Recombination between inverted repeats results in an inversion. **b**. Schematic representation of reciprocal duplications and deletions mediated by interchromosomal (left), interchromatid (middle) and intrachromatid (right) non-allelic homologous recombination (NAHR) using LCR pairs in direct orientation. Chromosomes are shown in black, with the centromere depicted by hashed lines. Yellow arrows depict LCRs. Letters adjacent to the chromatids refer to the flanking unique sequence (for example, A, a). Interchromosomal and interchromatid NAHR between LCRs in direct orientation result in reciprocal duplication and deletion, whereas intrachromatid NAHR only creates deletion. Signatures of homologous recombination include the sequence identity of the substrates (LCRs) used for NAHR, recombination hotspots within the LCRs, and evidence for gene conversion at the crossovers within the LCRs.

Due to their high degree of sequence identity, non-allelic copies of LCRs, instead of the copies at the usual allelic positions, can sometimes be aligned in meiosis or mitosis. This so-called 'misalignment' and the subsequent crossover between them can result in genomic rearrangements in progeny cells. The non-allelic copies thus act as the mediators (that is, substrates) of the homologous recombination and they are responsible for the observed breakpoint clustering. When the two LCRs are located on the same chromosome and in direct orientation, NAHR between them causes duplication and/or deletion. When they are on the same chromosome but in opposite orientation, NAHR results in inversion of the fragment flanked by them [2] (Figure 2a). NAHR between repeats on different chromosomes can lead to chromosomal translocation [2].

Evidence has shown that the strand exchanges during NAHR are not distributed evenly along the LCRs, but cluster in narrow 'hotspots' [7-10]. DNA structures capable of inducing double-strand breaks (DSB) (such as palindromes, non-B conformation DNA, minisatellites and DNA transposons) have often been found near the NAHR hotspots, indicating a potential link between NAHR and DSB [11,12]. At the same time, extensive linkage disequilibrium studies as well as detailed mapping of single loci clearly revealed that allelic homologous recombination (AHR) also has preferred hotspots [13-15].

Using sequencing-based approaches, De Raedt et al. [16] and Lindsay et al. [17] examined the fine structure of crossovers at the Neurofibromatosis type 1 (NF1; MIM162200) locus and Charcot-Marie-Tooth disease type 1A (CMT1A; MIM118220)/Hereditary Neuropathy with Liability to Pressure Palsies (HNPP; MIM162500) locus which often undergo NAHR. De Raedt et al. showed that NAHR hotspots can have strikingly similar positions in the LCR as the AHR hotspots in paralogous sequences [16]. Lindsay et al. found that in the same sequence fragment, NAHR hotspots can be located just adjacent to AHR hotspots and share similar properties of the distribution of strand exchanges [17]. These data provided evidence that the NAHR hotspots could be functionally closely related to AHR hotspots. Some of the NAHR and AHR hotspots still fall into the same regions in the current human genome; some of them may have overlapped in our ancestral genomes [16].

### 2. NAHR occurs in both meiotic and mitotic cells

NAHR in germ line cells leads to constitutional genomic rearrangements that can be manifested as genomic disorders [1,18]. Genomic disorders can be either inherited or sporadic, depending on whether the rearrangement was transmitted through the germ line or occurred *de novo *[19]. Prominent examples of inherited genomic disorders caused by NAHR include CMT1A and HNPP, caused by the recurrent duplication/deletion of a 1.4 Megabase (Mb) DNA fragment on chromosome 17p12; and sporadic genomic disorders include Potocki-Lupski syndrome (PTLS; MIM610883)/Smith-Magenis syndrome (SMS; MIM182290) caused by the reciprocal duplication/deletion on 17p11.2. The identification and detailed study of these rearrangements in patients has contributed significantly to our current knowledge on mechanisms of genomic rearrangements (the reports delineating the first recurrent disease-associated duplication rearrangement include [20,21], recently reviewed in [1,22]).

NAHR can also occur in mitosis, resulting in mosaic populations of somatic cells carrying genomic rearrangements. It is well appreciated that many cancers are related to somatic genomic rearrangements, some due to somatic NAHR [23,24]. Also, in blood cells of healthy persons elaborate PCR assays have been able to detect mosaic duplication, deletion [25,26] and inversion mediated by NAHR [27]. CNVs have been shown between monozygotic twins, highlighting the potential occurrence of genomic rearrangements in somatic cells [28]. Somatic NAHR can cause genomic disorders with mosaic manifestations, one example being the somatic NF1 deletions causing segmental neurofibromatosis [29]. Interestingly, Dempsey et al. reported a patient with mosaicism for both deletion del(22)(q11.2q11.2) and the reciprocal duplication dup(22)(q11.2q11.2), which were probably caused by a mitotic NAHR event early in embryogenesis [30].

The same pairs of LCRs can mediate both mitotic and meiotic NAHR events. The LCRs called REPA and REPB mapping in 17p11.2 are important mediators of somatic NAHRs leading to the formation of the dicentric isochromosome i(17q) in human neoplasia [31,32]; they also convey frequent meiotic NAHRs and cause this genomic locus to be highly variable in different populations [33]. Mitotic NAHR may not share the same hotspots with the meiotic NAHR mediated by the same pair of LCRs, as suggested by the observation of Turner et al. in their sperm-typing assay, where the primer pairs amplifying across the meiotic recombination hotspots in sperm DNA could not amplify any recombinant products from the DNA of blood cells [18]. The frequency of meiotic and mitotic NAHR on the same LCRs can be different as well [25]. Furthermore, the frequency and LCR usage of mitotic NAHRs could, theoretically, even vary among different somatic tissues although, to our knowledge, no data are currently available on this topic. Future studies using precise techniques to examine more loci should reveal further details on the similarities and differences between meiotic and mitotic NAHR.

### 3. Minimal efficient processing segments are required for efficient NAHR

For NAHR to take place, there must be segments of a minimal length sharing extremely high similarity or identity between the LCRs, named minimal efficient processing segments (MEPS). The importance of MEPS for intra- and interchromosomal mitotic recombination was demonstrated by Waldman and Liskay using mouse cell culture [34] and by Rubnitz and Subramani using monkey cell culture [35]. The placement of only two single-nucleotide mismatches, reducing the longest uninterrupted homology between two repeats from 232 to 134 base pairs (bp), resulted in a 20-fold reduction in intrachromosomal recombination [34]. Also, the frequency of interchromosomal recombination drops sharply when the homology was reduced from 214 to 163 bp [35].

The MEPS in human meiosis appear to be in the range of 300 to 500 bp in length, as empirically estimated from the analysis of the genomic rearrangements in CMT1A/HNPP patients [36]. The MEPS of mitotic NAHR may be different from meiotic NAHR. Steinmann et al. [29] identified nine somatic NF1 deletions conveyed by homology stretches shorter than 114 base pairs. Not all meiosis or mitosis events have the same demand of MEPS. With their single sperm/cell assay, Lam and Jeffreys [25] identified both meiotic and mitotic NAHR events between human alpha-globin genes mediated by matching fragments smaller than 50 bp [25]. The modest demand on MEPS in this case could be related to the proximity between the two NAHR substrate repeats. The distance between two LCRs is known to be one of the genomic architectural features that influence the efficiency of NAHR [2,5,37], and it has been observed that larger-sized genomic rearrangements, utilizing LCRs located further apart, often correlate with larger LCRs [2,5]. The repeats in the alpha-globin locus are only 5 kb away from each other, whereas the two LCRs of CMT1A/HNPP are separated by 1.4 Mb. Nevertheless, most of the rearrangements causing genomic disorders actually take place between LCRs which are 10 to 400 kb in length and have > 96% sequence identity [2,37]. The most frequent microdeletion syndrome, DiGeorge/Velocardiofacial (DG/VCFS; MIM188400, 192340) (frequency 1/4,000–1/8,000), is mediated by LCRs on chromosome 22q11.2, of 240 kb in length and sharing 99.7% sequence identity [38,39].

### 4. Reciprocal deletions and duplications do not occur at the same frequencies

The relative frequency of the reciprocal deletions and duplications from the NAHR events mediated by the same pair of LCRs is of both biological and clinical importance. In meiosis, NAHR can take place between paralogues on the same chromatid (intrachromatid), on sister chromatids (intrachromosomal or interchromatid) or on the homologous chromosomes (interchromosomal) [5,18]. Between two directly oriented LCRs, interchromatid and interchromosomal rearrangements result in reciprocal duplication and deletion, whereas intrachromatid rearrangements can only lead to deletion (Figure 2b). Thus, at least theoretically, the frequency of deletions should be always higher than duplications. The difference between the frequency of deletions and duplications reflects the frequency of intrachromatid NAHR.

The prevalence of several reciprocal duplication/deletion syndromes such as CMT1A/HNPP, PTLS/SMS, dup22(q11.2q11.2)/DG/VCFS has been used to estimate the relative frequency of duplications and deletions mediated by the same pairs of LCRs. The pitfall of these calculations is that one or even both events might be embryonically lethal or phenotypically mild so that the carriers will not be clinically ascertained. Furthermore, selection would occur in both germ cells and the organism, and may act differently on duplication versus deletion syndromes. To overcome these challenges, two groups took an experimental approach that used a single-sperm PCR assay to measure the duplication and deletion events directly. Turner et al. analyzed four NAHR loci related to well-studied genomic disorders (Williams-Beuren Syndrome deletion (WBS; MIM194050) and 7q11.23 duplication (MIM609757); the AZFa deletion (azoospermia (MIM415000) associated) and its reciprocal duplication; the HNPP deletion and CMT1A duplication; and the SMS deletion and PTLS duplication) in the sperm populations of five persons. Strikingly, they found that all five persons consistently displayed an approximately 2:1 ratio of deletion versus duplication in all three autosomal loci. For the AZFa locus on the Y-chromosome, the observed deletion versus duplication ratio 4:1 was even higher [18]. Thus, at least in meiosis, reciprocal duplications and deletions do not occur at equal frequency [18].

It is not clear how general this 'two deletions versus one duplication' rule is. Lam and Jeffreys [25,26] also performed single-sperm assays on the alpha-globin locus in two persons. One person showed the same deletion and duplication frequency, while the other person had a higher rate of duplications than deletions at this locus. This discrepancy between the two studies could be due to experimental design, as Turner et al. specifically measured the NAHR events across the so-called 'hotspots' in the LCR whereas Lam and Jeffreys observed the entire globin locus and could thus also record NAHR events outside the hotspots and other non-NAHR rearrangements. However, it could also reflect true differences among different NAHR loci, probably predisposed by the local genomic architecture (LCR length, distance of LCRs and so on).

Pedigree analysis of the haplotypes flanking the LCRs has often been used to differentiate between intra- and interchromosomal rearrangements [40-45]. These studies have revealed different findings for different syndromes. However, the haplotype assay has the limitation of being unable to differentiate between intrachromatid and interchromatid events, so the comparison can only be made between intrachromosomal (intra- plus interchromatid) and interchromosomal NAHR.

The single-sperm assay, however, allows the assessment of the intrachromatid events by observing the difference between deletion and duplication frequencies. Turner et al. [18] concluded that intrachromatid NAHR dominates at all hotspots they examined and the interchromatid NAHRs are very rare, with a frequency 50-fold lower than interchromosomal NAHRs. That the deletion versus duplication ratio at AZFa locus on Y chromosome is even higher than at the autosomal loci is likely because of the lack of interchromosomal NAHR.

The conclusion of Turner et al. agrees with the study of the WBS locus by Bayes et al. [44]. However, it awaits further confirmation from data at other loci before it can be accepted as a general rule. One should also bear in mind that this finding is only relevant for the NAHR events at the hotspots and does not describe any other types of rearrangements caused by different mechanisms.

### 5. NAHR can be different between males and females

There seem to be differences in NAHR frequency between male and female gametogenesis, as reflected by the different percentage of the two parental origins which were observed for several genomic disorders. The overwhelming majority of CMT1A duplications (nine in nine cases as reported in [46] and 26 in 28 cases in [47]) as well as 85% of spinal muscular atrophy (SMA; MIM253300) deletions [48] originate in spermatogenesis; whereas 80% of NF1 deletions are of maternal origin [8,49]. These apparent differences in maternally and paternally originated rearrangements might be due to intrinsic differences in NAHR between male and female germ lines, or might also reflect different selection bias against the rearranged allele between male and female germ lines, or a combination of both. Epigenetic modifications in male and female gametogenesis and gametes might contribute to both processes. The observed differences between male and female rearrangements do not seem to affect all NAHR loci to the same extent: for SMS/PTLS, no significant parental differences have been observed [50,51].

Whereas meiotic NAHRs causing genomic rearrangements either originate in or are inherited through the germ line of the previous generation, mitotic NAHRs occur in the somatic cells of the same individual who bears the rearrangements. It is intriguing that mitotic NAHR could also have a bias in females and males. Steinmann et al. [29] observed that 12 of their 13 segmental NF patients with deletions caused by somatic NAHR are females. The reason for this bias is not immediately obvious; it is not known if this bias is specific for the genomic locus or somatic tissues involved in the pathogenesis of NF, or whether it may reflect more general differences between male and female mitotic NAHR. Little data are available at the present time.

### 6. Using the NAHR mechanism to predict genomic disorders

The recognition of NAHR originated from the study of genomic disorders [2]. It is thus exciting that our now greater understanding of NAHR mechanisms, combined with bioinformatic analyses of the human genome, allows the prediction of regions prone to genomic instability, thus uncovering novel genomic disorders.

First, where recurrent deletions mediated by LCRs have been observed, we can confidently predict the occurrence of reciprocal duplication at the same sites, and *vice versa*. In recent years, with the application of mechanistic insight, we have witnessed the defining of the Potocki-Lupski syndrome as the predicted reciprocal rearrangement of SMS, dup(22)(q11.2) as the reciprocal rearrangement of DG/VCFS, and dup(7)(q11.23) as the reciprocal rearrangement for Williams-Beuren syndrome deletion [50-55]. The above-mentioned sperm-typing data of Turner et al. further confirmed the co-existence of the reciprocal rearrangements by experiments, while pointing out that the reciprocal syndromes can have unequal frequencies compared with the prevalence of the deletion syndromes.

Our lab has reported a 5 Mb uncommon but recurrent deletion in six SMS patients, which utilized alternative LCRs as NAHR substrates [56]. Although the reciprocal duplication of the common recurrent SMS deletion has been found in a number of cases and led to the definition of the PTLS syndrome, patients with the reciprocal duplication of the uncommon recurrent deletion have not yet been identified. It is thus of great interest that Turner et al. [18] did observe this duplication in their sperm assay, further underscoring the reciprocal nature of NAHR and affirming the anticipation that this duplication may also be found in patients. It should be pointed out that until now, we have only identified six uncommon recurrent deletions in our cohort of SMS patients; if the frequency of the reciprocal duplication is half that of the deletion, patients with the uncommon duplication should be even more rare.

Furthermore, the NAHR mechanisms based on LCRs have also led to the finding of a number of new genomic disorders. The majority of DG/VCFS patients have either a common 3 Mb or an atypical 1.5 Mb deletion on 22q11.2 mediated by LCR22-2 and LCR22-4, or LCR22-3a and LCR22-4, respectively ([38,54,57] and the references therein). The architecture of 22q11.2, however, also harbors additional LCR22s [38]. It was thus anticipated that recombinations mediated by other LCRs might also occur in this region. Indeed, using array comparative genomic hybridization (aCGH) techniques, Ben-Shachar et al. found six deletions mediated by LCR22-4, -5 and -6 [57]. These deletions are distal from the common DG/VCFS deletions and the patients have phenotypes overlapping with but distinct from DG/VCFS. These deletions were defined as the 22q11.2 distal deletion syndrome (MIM611867), a new genomic disorder [57]. The reciprocal duplications of these distal deletions have also been reported [54].

Also applying the principles of NAHR mediated by LCR, Sharp et al. [37,58] predicted microdeletion/microduplication rearrangements in new chromosomal loci that were previously not known to cause genomic syndromes. The authors [58] created a map of potential 'rearrangement hotspots' of the human genome, by localizing 130 sites of paired LCRs (SD) that are ≥ 10 kb in length, show ≥ 95% sequence identity and are separated by 50 kb to 10 Mb of intervening sequence [37]. A specific bacterial artificial chromosome (BAC) array was then designed including BAC clones interrogating each of these 130 NAHR candidate sites [37]. After ruling out the basal level of copy number polymorphisms in these sites by hybridizing a control population of 316 individuals [37], the authors analyzed the genomes of 290 idiopathic mental retardation patients and found deletions in four chromosomal loci (17q21.31, 1q21.1, 15q13, and 15q24) that are likely sites of recurrent rearrangements [58]. Three of the rearrangements were indeed identified as new microdeletion syndromes, with further cases found in other populations [58-60].

The microdeletion syndrome involving 17q21.31 was also identified by two other groups with a traditional systematic whole-genome array assaying individuals with idiopathic mental retardation [61-63]. In another study, the candidate NAHR loci array of Sharp et al. was used to assess 155 fetuses with congenital anomalies and identified a deletion involving 17q12 in a fetus with dysplastic kidneys [64]. They extended their study to include additional cohorts of patients and found that the deletion is also associated with congenital renal abnormalities and diabetes. The deletions are all in the range of under 1 Mb to 4 Mb in size, thus below the limit of the resolution of traditional cytogenetic detection [59,61,63].

Interestingly, the reciprocal duplication of the microdeletion in 17q12 (mediated by the same LCRs) was identified in two individuals with mental retardation and/or epilepsy [64]. The reciprocal duplication of the 15q13 deletion has also been identified in a healthy person [59] and the reciprocal duplication of the 17q21.31 deletion was reported in a patient with phenotypes including severe psychomotor developmental delay and facial dysmorphism [65]. The duplications corresponding to the remaining microdeletions will probably also be identified soon, although it is not known yet what kind of phenotypes will be related to them.

## Some simple non-recurrent rearrangements can occur via NHEJ

NHEJ is one of the two major mechanisms used by eukaryotic cells to repair DSB and has been described in organisms from bacteria to mammals [66-68]. NHEJ is routinely utilized by human cells to repair both 'physiological' DSBs, such as in V(D)J recombinations, and 'pathological' DSBs, such as those caused by ionizing radiation or reactive oxygen species. Inherited defects in NHEJ account for about 15% of human severe combined immunodeficiency (SCID) [69]. NHEJ is also currently considered to be the major mechanism rejoining translocated chromosomes in cancer [70].

**Figure 3:**
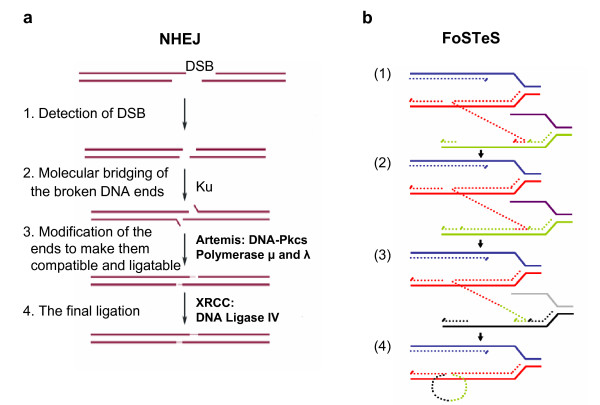
**Genomic rearrangement mechanisms**. **a**. (Adapted from [66]) Non-homologous end-joining (NHEJ) in vertebrates. A double-stranded DNA break (DSB) occurs and is repaired via NHEJ mechanism. The two thick lines depict two DNA strands with DSB, the thin segments in the middle represent the modifications which the ends have gone through before the final ligation. The enzyme machineries catalyzing each step are briefly summarized. They are described in details in references [65] and [70]. Note at step 3 that in order to repair ends, some addition or deletion of bases may be required, leaving behind a 'signature' of NHEJ. **b**. (Adapted from [82]) After the original stalling of the replication fork (dark blue and red, solid lines), the lagging strand (red, dotted line) disengages and anneals to a second fork (purple and green, solid lines) via microhomology (1), followed by (2) extension of the now 'primed' second fork and DNA synthesis (green, dotted line). After the fork disengages (3), the tethered original fork (dark blue and red, solid lines) with its lagging strand (red and green, dotted lines) could invade a third fork (gray and black, solid lines). Dotted lines represent newly synthesized DNA. Serial replication fork disengaging and lagging strand invasion could occur several times (e.g. FoSTeS x 2, FoSTeS x 3, ... etc.) before (4) resumption of replication on the original template.

NHEJ proceeds in four steps (Figure 3a): detection of DSB; molecular bridging of both broken DNA ends; modification of the ends to make them compatible and ligatable; and the final ligation step [68]. This process determines the two important characteristics of NHEJ: first, neither LCRs nor MEPS are obligatorily required for NHEJ; and second, NHEJ leaves an 'information scar' [71] at the rejoining site as the pre-rejoining editing of the ends includes cleavage or addition of several nucleotides from or to the ends [71].

Nobile et al. and Toffolati et al. [72,73] sequenced the breakpoints of 19 patients with muscular dystrophy due to non-recurrent deletions in introns 47 and 48 of the *DMD *gene. These deletions were not flanked by LCRs and the junctions showed microhomology (2 to 4 nucleotides) in seven cases, short insertions (1 to 5 nucleotides) in three cases and short duplications of surrounding fragments up to 25 bp in three cases. Other junctions either contained short sequences of unknown origin or did not show any microhomology, which might be due to the editing process in NHEJ. These events thus fit well with the features of the NHEJ mechanism. Remarkably,16 of the 38 (42%) breakpoints in these two publications fell within repetitive elements such as LTR, LINE, *Alu*, MIR and MER2 DNA elements; also, sequence motifs known to be capable of causing DSB or curving DNA, such as TTTAAA, are present in proximity to many of these junctions [72,73].

Inoue et al. identified two apparently NHEJ-mediated deletions of the *PLP1 *(proteolipid protein) gene in Xq22 in patients with Pelizaeus-Merzbacher disease (PMD; MIM312080). Breakpoint analysis showed 12 base pair and 34 base pair sequences of unknown origin at the junction [74]. Interestingly, the distal breakpoints of both deletions were located in a 32 kb LCR termed LCR-PMDB [74]. Shaw and Lupski reported two non-recurrent SMS deletions apparently caused by NHEJ; the proximal breakpoints of both deletions are localized in an LCR (the proximal SMS-REP) [75]. One of them occurred within a MER5B transposon element in the SMS-REP, while the other was located in proximity to a MIR3 element and an L2 LINE sequence. The distal breakpoint of the latter deletion was localized between an LIMC4 LINE element and an *Alu*Sc element [75]. Many breakpoints of 17p translocations and other unusual-sized deletions also occurred within LCRs [76]. Consistent with the finding of repetitive and DNA breaking elements at the NHEJ breakpoints by Toffolatti et al. and Nobile et al., the locations of the *PLP1 *deletions and SMS deletions as well as the 17p translocation and deletion breakpoints map within the LCRs and are close to other repetitive DNA elements. These findings suggest that although NHEJ is not directly mediated by nor strictly dependent on certain genomic architectural elements in the way that NAHR is dependent on LCRs, it may still be stimulated and regulated by the genomic architecture [4,76].

Combined with the DSB homologous repair (HR) as a two-step mechanism, NHEJ was also used to explain duplications [77,78]. Woodward et al. and Lee et al. observed non-recurrent duplications in the *PLP1 *region in the majority of PMD patients; these duplications are non-recurrent although some of them do show breakpoint grouping (not clustering) at one end (Figure 1c) [77,78]. Most of the duplications are tandem in orientation. Padiath et al. observed similar non-recurrent tandem duplications in the *LMNB1 (*coding for Lamin B1) region in subjects with autosomal dominant leukodystrophy [79]. The junctions sometimes show microhomology [77,79], and sometimes have insertions of one to six nucleotides [77,78]. Woodward et al. and Lee et al. proposed that in the first step of the rearrangement, a single DSB occurred in one strand; one of the broken ends then invaded and copied from the sister chromatid and caused the duplication. The ends were then rejoined via NHEJ [77,78].

## A DNA replication-based mechanism FoSTeS can account for complex genomic rearrangements

The study of rearrangement mechanisms obviously benefits from the development of new techniques to observe the rearrangements and breakpoints with a higher resolution. In the past, fluorescence *in situ *hybridization (FISH) has defined the duplications and deletions with resolution to about one BAC clone (150 to 200 kb) and accelerated the discovery of NAHR and NHEJ mechanisms. Recently, the advent of array-based CGH [reviewed in [80,81]] has provided an unprecedented ability to observe the often complex details of genomic rearrangements, and has led to the proposal of the DNA replication-based FoSTeS model as the third major mechanism for human genomic rearrangements [82].

**Figure 4:**
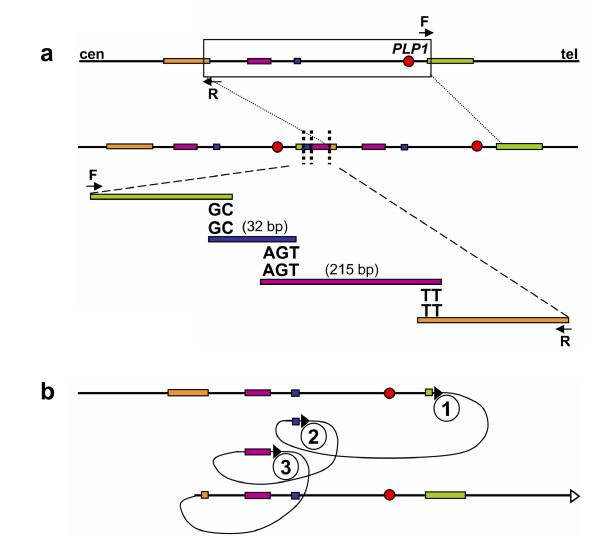
**One Pelizaeus-Merzbacher disease (PMD)-associated complex *PLP1 *rearrangement results from multiple FoSTeS events, FoSTeS × 3 (Adapted from **[82]**)**. **a**. Duplication junctions (vertical dotted lines) for one PMD patient are displayed relative to reference sequence, with the duplicated region boxed. Two or three base pairs of microhomology were found at the breakpoint junctions (i.e. "joint points") after amplification with outward-facing primers (F and R). **b**. Illustration of the order, origins, and relative orientations of junctional (pink and blue) and boundary reference sequences (orange and green) for the PMD patient. Arrowheads show direction of DNA relative to the positive strand; filled arrowheads with circled numbers below represent a FoSTeS event; open arrowhead marks resumption of replication on the original template. Proximal (centromeric) and distal (telomeric) are in relation to *PLP1 *(red circle).

Lee et al. used a 44 K Agilent custom array to study the genomic region surrounding *PLP1 *in PMD patients [82]. This array, with resolution of almost two interrogating oligonucleotides each kb, enabled the observation of non-recurrent rearrangements in PMD patients that were more complicated than simple duplication or deletion. The apparent duplications initially observed by FISH are often actually interrupted by triplicated or deleted fragments, or fragments with normal copy numbers. Subsequent mapping of breakpoints revealed further complexity of these rearrangements by showing that some of the fragments are inverted or translocated to another region. Microhomology of two to five nucleotides was found at each sequenced breakpoint junction [82]. One of the PMD cases resulting from FoSTeS-mediated complex rearrangement of the *PLP1 *locus is shown in Figure 4.

**Figure 5:**
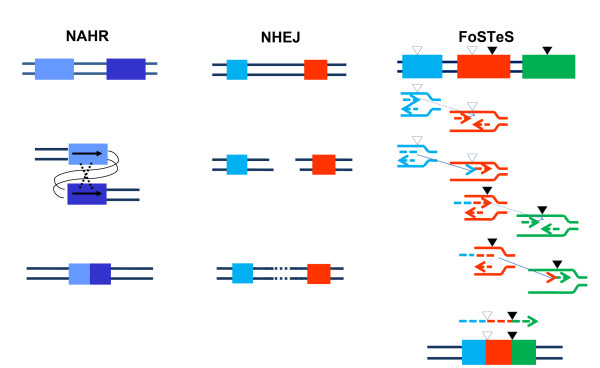
**Comparison of non-allelic homologous recombination, non-homologous end-joining and Fork Stalling and Template Switching mechanisms resulting in genomic duplication/deletion**. The two thin lines in all three schemes represent the double strands of DNA. Left column: An intrachromatid non-allelic homologous recombination (NAHR) event. Rectangles in different shades of blue depict two directly orientated low-copy reapeats (LCRs) sharing high homology (97% to 98%), which align at non-allelic rather than allelic positions and the subsequent recombination causes deletion or duplication (reciprocal events but not with equivalent frequencies) of part of the two LCRs as well as the segment flanked by them. Middle column: a non-homologous end-joining (NHEJ) event. Double-strand breaks (DSBs) are created between the two sequences represented as a blue and a red rectangle with no homology between each other. The NHEJ system modifies and rejoins the two ends, resulting in the deletion of the segment between the two DSBs. Right column: a Fork Stalling and Template Switching (FoSTeS) × 2 event causing a complex deletion involving two fragments. No extensive homology is required between the substrate sequences depicted by a blue, a red and a green rectangle. However, the small open triangle heading downwards depicts a site bearing microhomology (2 to 5 base pairs) between the blue and the red sequences, and the small filled triangle heading downwards depicts another site bearing microhomology between the red and the green sequences. Different from NAHR and NHEJ, the FoSTeS event occurs during DNA replication. The replication forks from the two surrounding sequences are shown in the same color as the rectangles. The leading nascent strand at the left side (blue or red) fork invades the right side (red or green) fork via the demonstrated microhomology, and primes its own further synthesis using the right side fork as template. This event happens twice, causing deletion of the two fragments flanked by each pair of microhomology sites. Note the juxtaposition of genomic sequences from multiple distinct regions yielding complex rearrangements.

It is difficult to explain this complexity by either the NAHR or NHEJ recombination mechanisms. Inspired by the findings in *Escherichia coli *[83], Lee et al. proposed the replication Fork Stalling and Template Switching (FoSTeS) Model (Figure 3b). According to this model, during DNA replication, the DNA replication fork stalls at one position, the lagging strand disengages from the original template, transfers and then anneals, by virtue of microhomology at the 3' end, to another replication fork in physical proximity (not necessarily adjacent in primary sequence), 'primes', and restarts the DNA synthesis [82]. The invasion and annealing depends on the microhomology between the invaded site and the original site. Upon annealing, the transferred strand primes its own template-driven extension at the transferred fork. This priming results in a 'join point' rather than a breakpoint, signified by a transition from one segment of the genome to another – the template-driven juxtaposition of genomic sequences. Switching to another fork located downstream (forward invasion) would result in a deletion, whereas switching to a fork located upstream (backward invasion) results in a duplication. Depending on whether the lagging or leading strand in the new fork was invaded and copied, and the direction of the fork progression, the erroneously incorporated fragment from the new replication fork would be in direct or inverted orientation to its original position. This procedure of disengaging, invading/annealing and synthesis/extension could occur multiple times in series (that is, FoSTeS × 2, FoSTeS × 3, and so on) (Figure 5), likely reflecting the poor processivity of the involved DNA polymerase, and causing the observed complex rearrangements.

Array CGH data on several other genomic regions, including the SMS/PTLS locus [50,84] (Lupski Lab, manuscript in preparation) and the *MECP2 *locus [85-88] have confirmed the complex nature of many other non-recurrent rearrangements, some of which were thought to be simple deletion or tandem duplication before the oligoarray technique was available. Likewise, the FoSTeS mechanism can potentially explain some of the complex rearrangements observed at the *DMD *locus [89]. The FoSTeS model is currently the only major rearrangement mechanism that could explain these complex rearrangements. Furthermore, some complex chromosome rearrangements (CCR) unveiled by recent cytogenetic data can also be explained by FoSTeS [84]. Intriguingly, some tandem duplications in the *PLP1 *and *LMNB *region [77,79] which were previously explained by a model combining HR and NHEJ, especially those with microhomology at the junction [77,79], can be more parsimoniously explained by the FoSTeS model including the strand switching template only once (FoSTeS × 1).

Interestingly, similar to the *PLP1 *region, the SMS/PTLS and *MECP2 *regions were also found to have very complex genomic architecture with multiple LCRs [1,85,86]. These LCRs, although they do not mediate FoSTeS directly, might be able to bring replication forks together to facilitate the replication fork switching event. Furthermore, highly enriched *Alu *repeats and high GC-content sequences were observed in proximity to the *MECP2 *complex recombination region [85]. So, like NAHR and NHEJ, FoSTeS is probably also influenced by the local genomic architecture. Unlike NAHR or NHEJ, FoSTeS rearrangement is currently based on the translocation of the end of a single nascent strand, so the genomic architectures facilitating FoSTeS may function via a mechanism that does not involve DSB intermediates. Nevertheless, a microhomology-mediated break-induced replication (MMBIR) model has also been proposed, in which the rearrangement is initiated by a single-end double-strand DNA break resulting from a collapsed replication fork (Hastings et al. personal communication). As more and more sophisticated array techniques are being used in more and more laboratories, we look forward to the discovery of more complex rearrangements and using them to further verify and modify the current FoSTeS model.

## Some gross genomic rearrangements and small-scale gene mutations might share similar mechanisms

**Figure 6:**
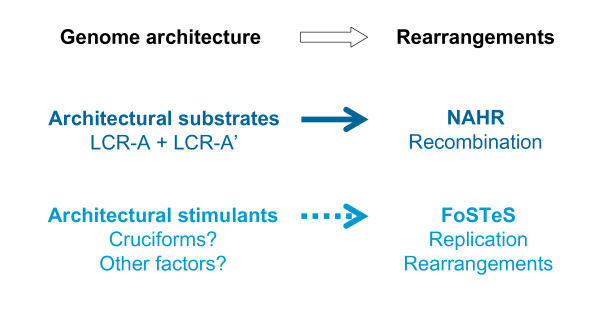
**Genomic architecture is crucial for the genomic rearrangements**. The low-copy repeats serve as substrates and thus are an indispensable requirement of non-allelic homologous recombination. Current data suggest that local genomic architecture, including palindromes or cruciforms, might be a stimulus for the Fork Stalling and Template Switching (FoSTeS) rearrangement as well, although these architectural elements are not necessarily directly involved in the FoSTeS rearrangement *per se*. This could account for the observation of breakpoint grouping with non-recurrent rearrangements at some loci.

The most significant difference between FoSTeS and the other two rearrangement mechanisms (NAHR, NHEJ) is that it is a replication-based mechanism; the rearrangement is induced by errors in the replication procedure. It has been thought that small monogenic genetic mutations often reflect errors of DNA replication and/or repair [4], whereas genomic rearrangements are thought to be caused by other mechanisms induced by or associated with structural features (genomic architecture) of the local genomic region [1]. The FoSTeS mechanism suggests that large genomic rearrangement involving thousands or even millions of DNA base pairs can be due to replication errors as well, perhaps also stimulated by local genome architecture such as cruciforms (Figure 6).

Chen and colleagues [90-93] studied the breakpoints of 'smaller' DNA rearrangements (between 21 bp and up to 10 kb) including duplications, deletions, insertions, and inversions collected in the Human Gene Mutation Database (HGMD) [94]. They found that many of them have a complex nature (similar to the complex nature of the 'large' rearrangements now being observed using array CGH), instead of being simple duplications and deletions. They proposed the serial replication slippage (SRS) model to explain these complex gene mutations. The SRS model is an extension of the classical replication slippage model [95]; it assumes that the 3' end of the nascent strand could dissociate from the original template and invade other templates on the basis of microhomology. Depending on whether the strand slippage occurs forwards or backwards, the nascent strand will have a deletion or duplication. Making use of reversed repeats, the nascent strand can also invade in the reverse orientation and thus incorporate an inverted segment. The slippage can happen serially, creating the complex rearrangements Chen et al. observed of small sizes between 21 bp and several kb.

The SRS model proposed for small gene mutations shares some general features with the FoSTeS model proposed for the larger rearrangements. Both models assume serial replication slippage, and both stress the importance of the genomic architectural elements such as palindromic DNA, stem-loop structures, repeats and so on, which may facilitate the initial stalling of the replication fork. While the SRS model assumes that replication slippage occurs on closely adjacent sites (possibly inside the same replication fork) and causes DNA rearrangements of small sizes, the FoSTeS model emphasizes that the template switch can occur over long distances (120 kb to 550 kb observed to date) to another replication fork (given the spatial closeness of the two forks) and cause DNA rearrangements on a much larger scale. Furthermore, FoSTeS × 1 could explain deletion and duplication events previously proposed to occur via NHEJ, in a way similar to the explanation of small deletions and duplication using the SRS model; the observed microhomology at the join point reflecting the priming event rather than a recombination/repair process. It is interesting to realize that although we have been talking about monogenic (often small) and genomic (often large) rearrangements in different contexts, some of them apparently have similar complexity and might be caused by very similar mechanisms.

## Conclusion

NAHR was the first major DNA rearrangement mechanism identified to cause genomic disorders. NAHR occurs during both meiosis and mitosis and it requires two LCRs with sufficient length of high homology to act as recombination substrates (Figures 2 and 6). Based upon the principles or 'rules' elucidated by studies of this mechanism, new genomic disorders have been successfully predicted and uncovered. Although this LCR-based prominent theme of NAHR remains the same, recent research has shown that some details of NAHR mechanism, such as the frequency of the recombination and the length requirement of homology between the LCRs, can differ between males and females and between meiosis and mitosis.

NHEJ and FoSTeS were later employed to explain other genomic rearrangements. Both models are still awaiting more data for further elucidation and modification. FoSTeS is a unique mechanism compared with NAHR and NHEJ, especially in that it is a replication-based rearrangement pathway and does not necessarily rely on the pre-formation of DSB. Although still very limited, our preliminary data imply that FoSTeS might be a major mechanism for duplication CNV and thus a major driver of the Ohno 'gene duplication/divergence' evolutionary hypothesis [96]. Indeed, FoSTeS might also have been the driving force in the origin of the LCRs in the human genome. It is well known that DNA polymerases have an intrinsic error rate leading to base substitution, a fact which is central to genome stability, disease origins and evolution of species. It is tempting to speculate that there may be an endogenous polymerase error rate for FoSTeS as well, analogous to the base substitution error rate. A related question would be whether or not disorders that are frequently sporadic and occur via FoSTeS are associated with advanced paternal age, as are point mutations that are due to DNA replication errors [19]. It has been proposed that carriers of hereditary non-polyposis colon cancer (HNPCC, MIM120435) with mutations in genes involved in the DNA mismatch repair pathway may be more susceptible to somatic genome rearrangements caused by NAHR events [97]. One could also hypothesize that some other individuals could be more prone to genomic rearrangements mediated by FoSTeS because of mutations/functional polymorphisms in the DNA replication machinery.

It has been clearly shown that both NHEJ and FoSTeS can be indeed stimulated by local genomic architecture, but no direct association of specific DNA elements with either model (such as LCRs associated with NAHR) has been experimentally identified. It is an interesting question to which degree NHEJ and FoSTeS are structurally determined or enhanced by specific genome architecture and whether some day we may be able to predict regions of human genome instability caused by NHEJ and FoSTeS events, as we have predicted NAHR events and the related genomic disorders. Currently limited data suggest that a palindrome or cruciform may stimulate FoSTeS (Figure 6).

There are still many unsolved, exciting questions regarding the mechanisms of human genomic rearrangements in general. Evidence is emerging that genomic rearrangements, despite their likely common basic mechanisms, might be differently regulated between germ line and somatic cells, between embryogenesis and adulthood, and between cancer cells, stem cells, and differentiated cells [98,99]. It is well known that other genome activities (such as transcription) can be fundamentally different in different cellular settings. It is thus tempting to relate the differences in genomic arrangements within these developmental contexts and cellular environments to the differences of other genome-involving processes, and to ask the question of whether there is an interaction or some kind of crosstalk between genomic rearrangement and other cellular processes. We know that NHEJ rearrangements are physiologically relevant in generating antibody diversity [66]; are there other 'programmed' rearrangements including inversions [27] which are employed in the development or regulation of other biological events? Finally, are there other mechanisms for genomic rearrangements in addition to the three discussed in this review?

For the latter question, some data are starting to emerge from two genome-wide structural variation studies. Korbel et al. [100] and Kidd et al. [101] used the paired-end-mapping (PEM) [100] and the fosmid-based end-sequencing-pair (ESP) [101] methods respectively, to systematically identify structural variants (SVs) in human genomes. Korbel et al. identified 1297 SVs including 853 deletions, 322 insertions and 122 inversions, and sequenced the breakpoints of 188 SV indels and 14 inversions. It is very interesting that almost all of the SVs bear signatures of either NAHR (surrounded by LCRs or repetitive sequences such as SINEs, LINEs), NHEJ or FoSTeS (microhomology at the junction), or retrotranspositions (mostly L1 elements). (Retrotransposition causes rearrangements in the genome via RNA-mediated mechanisms and is not the subject of this review.) Very few SVs do not fall into any of the three categories (Korbel, personal communications). Kidd et al. inferred mechanisms from breakpoints analysis for 227 SV indels and 34 inversions, and similarly identified evidence for NAHR, NHEJ or FoSTeS mechanisms. There are differences between the results of the two papers. The calculated ratio of NAHR-mediated events in SV indels, for example, is 14% according to Korbel et al., but much higher (39%) in Kidd et al. These differences may be due to the differences in their methodology or design; that of Kidd et al. is likely more efficient in detecting larger variations. Nevertheless, it seems that the three major rearrangement mechanisms – NAHR, NHEJ and FoSTeS – can explain the majority of the DNA rearrangements occurring in our genomes.

It is also of interest that the sequence analysis of both studies indicated that a portion of NAHR events utilize repetitive elements (SINEs, LINEs, LTRs), rather than LCRs as homology substrates. This finding is consistent with our previous data [75] showing that some non-recurrent deletions of SMS patients can be mediated by NAHR between *Alu *sequences. These *Alu*s are from the evolutionarily youngest subfamilies *Alu*S and *Alu*Y, and share a high degree of homology with each other. This homology apparently fulfills the conditions for MEPS and is enough to enable occasional non-allelic homology mediated recombination between two *Alu *sequences. However, the length of homology between two *Alu *sequences is much shorter than that between two usual LCRs, which may explain the lower frequency of the *Alu*-mediated recombination events than the LCR-mediated NAHRs.

Both PEM and ESP are based on the sequencing of small fragments (~3 kb for PEM and up to 40 kb for ESP) of the individual genomes and then comparing the distance between both ends of the fragments with the value of the reference genome. It should be noted that large duplications that can not be spanned by these small fragments might be underrepresented in the SVs identified by PEM and ESP because of the design of the methodology. Furthermore, these approaches: (i) may not readily detect complex genomic rearrangements, and (ii) the computational "filtering" accompanying the match of shotgun and short sequence reads to the reference genome may result in lack of identification of breakpoint sequences. On the other hand, this strategy is very powerful in identifying DNA sequence read information at the breakpoints of the deletion and inversion SVs. Future developments of even more sophisticated and sensitive genome-wide assay technologies will provide a more extensive overview of the structural variants in our genome and greatly facilitate the research on the mechanisms for CNV and other genomic rearrangements.

## Competing interests

The authors declare that they have no competing interests.

## Authors' contributions

WG and JRL wrote the review manuscript. FZ participated in the discussion and helped to edit the figures. All authors read and approved the final manuscript.
